# A Review of The Application of Spectroscopy to Flavonoids from Medicine and Food Homology Materials

**DOI:** 10.3390/molecules27227766

**Published:** 2022-11-11

**Authors:** Lin Zou, Huijun Li, Xuejie Ding, Zifan Liu, Dongqiong He, Jamal A. H. Kowah, Lisheng Wang, Mingqing Yuan, Xu Liu

**Affiliations:** 1 College of Medicine, Guangxi University, Nanning 530004, China; 2 College of Chemistry and Chemical Engineering, Guangxi University, Nanning 530004, China

**Keywords:** flavonoid, medicine and food homology, spectroscopy

## Abstract

Medicinal and food homology materials are a group of drugs in herbal medicine that have nutritional value and can be used as functional food, with great potential for development and application. Flavonoids are one of the major groups of components in pharmaceutical and food materials that have been found to possess a variety of biological activities and pharmacological effects. More and more analytical techniques are being used in the study of flavonoid components of medicinal and food homology materials. Compared to traditional analytical methods, spectroscopic analysis has the advantages of being rapid, economical and free of chemical waste. It is therefore widely used for the identification and analysis of herbal components. This paper reviews the application of spectroscopic techniques in the study of flavonoid components in medicinal and food homology materials, including structure determination, content determination, quality identification, interaction studies, and the corresponding chemometrics. This review may provide some reference and assistance for future studies on the flavonoid composition of other medicinal and food homology materials.

## 1. Introduction

In recent years, as people have become more conscious of food safety and health, more and more people are looking at foods with health benefits and therapeutic effects. In Chinese traditional medicine concepts, this is referred to as “medicine and food come from the same source” or “medicinal and food homology”. In the earliest records of the Yellow Emperor’s Internal Classic, food can be used as a complementary function to medicine to treat disease. In the Tang dynasty, the “Qianjin recipe”, and then in the Yuan dynasty, the “principles of correct diet”(Yin Shan Zheng Yao), the efficacy and contraindications of food were documented, and the theoretical system of the medicinal and food homology was gradually improved [1]. In November 2021, China’s National Health and Wellness Commission issued the “Regulations on the Management of the Catalogue of Substances that are Traditionally Both Food and Chinese Medicinal Herbs”, listing 110 medicinal and food homology materials [2].

These herbs, which have been popular in China for millennia, possess high medical value. Unfortunately, however, their clinical development remains highly limited. An important influencing factor is that the composition of herbal medicines is often not a single but a complex mixture, thus making quality control of herbal medicines more difficult. Therefore, qualitative identification and quantitative analysis of herbs is essential to ensure quality standards and safe efficacy. For medicinal and food homology materials, it is even more important to carry out quality assessments to ensure safety due to the particular characteristics of the materials that differ from those of ordinary food products. The Pharmacopoeia of the People’s Republic of China and the Law of the People’s Republic of China on the Administration of Medicinal Products have strict regulations on medicinal and food homology materials.

**Figure 1:**
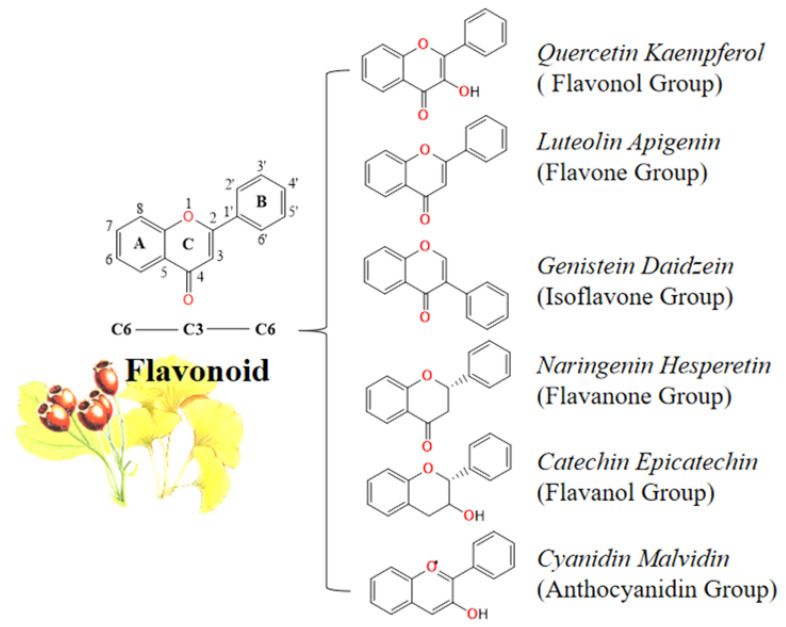
Classification of flavonoids.

Secondary metabolites in medicinal plants include flavonoids, polysaccharides, terpenoids, quinones, steroids and others, of which flavonoids are one of the most important active ingredients [3]. Flavonoids are a large class of polyphenols, which are widely present in various plants in free form or as glycosides. The basic skeletal structure of flavonoids consists of two benzene rings (A and B) which are linked by an oxygen-containing heterocyclic pyran ring (C) [4]. Flavonoids can be divided into different six major groups depending on the unsaturation of the linking chain, the connection between the rings and the chemical structure, including flavanones, isoflavonoids, flavanols, flavonols, flavones and anthocyanidins [5] (Figure 1).

**Figure 2:**
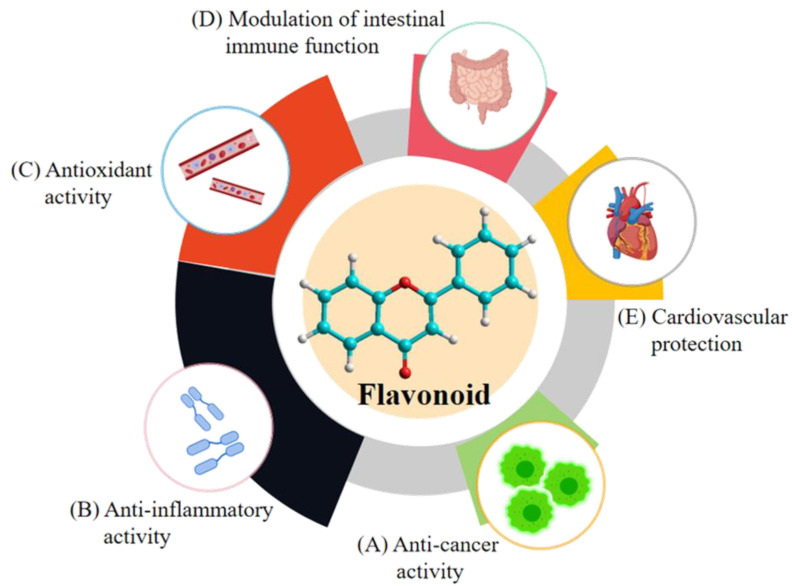
Pharmacological activities of flavonoids.

Flavonoids derived from food and herbs have been found to contain a variety of pharmacological activities, such as antioxidant [6], anti-inflammatory [4], anti-cancer [5,7], modulation of intestinal immune function [8] and cardiovascular protection [9] (Figure 2).

The flavonoids extracted from various herbal medicines are numerous and have complex and diverse structures. Therefore, a range of analytical technical tools and methods, such as high-performance liquid chromatography (HPLC), gas chromatography (GC), mass spectrometry (MS) and spectroscopy, are used to determine the basic structure and characterization of flavonoid components. Traditional analytical techniques, such as HPLC [10], GC [11], liquid chromatography-mass spectrometry (LC-MS) [12,13], gas chromatography-mass spectrometry (GC-MS) [14], have been used to determine the concentration as well as identify the structure of the substance. However, these methods are expensive, time-consuming, require complex operation skills and consume large amounts of solvents and reagents. In addition, these methods usually measure a small number of samples to represent a larger sample, so it is not suitable for large-scale measurement applications. At present, various spectroscopic techniques are frequently used for the detection and identification of biological molecules, such as UV, NIR, fluorescence spectroscopy, terahertz time-domain spectroscopy, etc.

Compared to these techniques, spectroscopic analysis has the advantage of being relatively simple, inexpensive and does not require extended preparation methods of samples or other chemicals [15]. On the other hand, because each compound has its own specific characteristic spectral information, it is easy to differentiate classes of functional groups, the structure of bonds within compounds and their conformation by corresponding spectral profiles, which has led to the widespread use of spectroscopic analysis for the development and study of flavonoid components [16].

This paper provides a review of spectroscopic techniques used in recent years in the quality control and development applications of flavonoid in medicinal and food homology materials and details the advantages and research progress of each technique. These findings provide an important scientific basis for future research and development of flavonoid components in medicinal and food homology materials.

## 2. Qualitative and Quantitative Analysis

The analytical identification of active compounds extracted from herbal medicines also depends on various analytical techniques. The detection of flavonoids in herbal medicines has received increasing attention. Many studies related to the qualitative and quantitative analysis of flavonoid components in medicinal and food homologous materials have been published. Compared with the traditional time-consuming, labor-intensive and expensive chromatography methods, the spectroscopic method is simple, fast and environmentally friendly [17]. Spectral analysis is based on their characteristic spectra and is used to study the structure of compounds or to determine their chemical composition [18]. Various organic compounds with different structures have their own unique characteristic spectra. Table 1 has summarised the spectroscopic techniques used for quantitative and qualitative analysis of medicinal and food homology materials.

### 2.1. Nuclear Magnetic Resonance

Nuclear magnetic resonance (NMR) spectroscopy can detect and quantify molecular interactions to accurately characterize the structure of flavonoid molecules in complex samples, although it has disadvantages such as high cost and may not be suitable for all applications [41]. Duan et al. isolated and purified 21 compounds from the Platycladi Cacumen extract and identified the chemical structures of several flavonoids by NMR [19]. Shang et al. studied the chemical composition and biological activities in four different parts of the roots, stems, leaves and seeds of *Glycyrrhiza uralensis* [20]. Three flavonoid C-glycosides were identified by NMR spectroscopic analysis. Bao et al. isolated 14 isoflavone components from the root of *Astragalus membranaceus*, the planar structures of which were determined by detailed analysis of 1D and 2D NMR data [21]. Xie et al. first analyzed four major flavonoids in *Capsella bursa-pastoris* (L.) by UPLC, 1H-NMR and 13C-NMR spectroscopic techniques to investigate the effect of *Capsella bursa-pastoris* (L.) on the clinical treatment of cataracts [22]. Wang et al. isolated and identified four new compounds and eight known flavonoids from the leaves of *Glycyrrhiza uralansis* and determined their structures by high-resolution electrospray mass spectrometry (HR-ESI-MS) and UV spectrum and 1H-nuclear magnetic resonance (NMR) and 13C-NMR spectroscopy [23]. Yi et al. determined the 1H and 13C NMR chemical shifts and NMR shielding parameters of daidzein and puerarin, the major flavonoid active ingredients in Radix puerariae, by NMR analysis [24]. Xu et al. reported 109 chemical constituents, including 21 flavonoids, 6 flavones and 35 isoflavonoids, from the mature fruit of *Psoralea corylifolia* L. The structures of the compounds were elucidated by 1H NMR, 13C NMR, 2D NMR and Rh2(OCOCF3)4 and Mo2(OAc)4-induced circular dichroism spectroscopic methods [25].

### 2.2. Terahertz Time-Domain Spectroscopy

Terahertz (THz) spectroscopy is an emerging and powerful investigation technology that contains abundant information about the physics, chemistry and structure of materials. In contrast to conventional far-infrared spectroscopy, terahertz spectroscopy exploits a part of the electromagnetic spectrum that lies between the microwave and infrared regions. In the terahertz band, biological molecules conduct complex molecular vibrations such as rotations, low frequency bond vibrations, hydrogen bonds and van der Waals forces. Based on terahertz characteristic spectra, biomolecules can be identified effectively, especially for those with similar chemical structures. In recent decades, terahertz spectroscopy has been widely used in the fields of physics, chemistry, materials science and biomedicine on account of its rapid, safe and non-destructive advantages [42,43]. However, terahertz spectroscopy also has limitations in terms of limited penetration, scattering effects and limited sensitivity [44]. Furthermore, the cost of terahertz spectroscopy is still high compared to other applications and the current terahertz database on natural active compounds is incomplete and needs further improvement and development.

Yin et al. used terahertz time-domain spectroscopy (THz-TDS) for the qualitative identification and quantitative analysis of ten common flavonoids, including baicalein, baicalin, apigenin, quercetin, naringenin, hesperetin, daidzein, genistein, puerarin, and gastrodin [26]. These flavonoids with similar molecular structures had significantly different characteristic absorption peaks in the terahertz band and were distinguished by the terahertz absorption spectrum. Yan et al. identified three flavonols with similar structures, including myricetin, quercetin, and kaempferol, and determined their concentrations by terahertz spectroscopy [27].

### 2.3. Fluorescence Spectroscopy

Fluorescence spectroscopy is now increasingly used for quality control and monitoring analysis of food and pharmaceuticals as a sensitive, simple and fast detection technique [45]. Flavonoid components will produce fluorescence when exposed to constant excitation light due to their native fluorescence properties. Shan et al. used a fluorescence spectrophotometer to obtain synchronous fluorescence spectra of tea infusion to quantify the content of flavonoids in green tea. The excitation/emission spectra of flavonols were in the range of 365–390 nm/450–470 nm and those of flavanols were in the range of 480–500 nm/510–520 nm [46]. In recent years, as nanotechnology becomes more sophisticated, various new nano-fluorescent sensor-based fluorescence spectroscopy has found more applications in quality assessment [47]. Lan et al. used a nanomaterial-based fluorescent sensor combined with spectral splicing to successfully evaluate the quality of *Citri Reticulatae Pericarpium* and identify their storage year [28]. Specifically, nanogold particles and cadmium telluride quantum dots were chosen as nanosensors and mixed with aqueous extracts of *Citri Reticulatae Pericarpium* to collect fluorescence quenching spectra. Then, the self-fluorescence and fluorescence quenching spectra of the same sample were combined to integrate the spectra of different fluorescence sensing systems at the same coordinate axes to obtain spliced spectra. This new strategy achieved accurate recognition of different *Citri Reticulatae Pericarpium* samples by identifying the interaction between the nanoparticles and the fluorescent components in the *Citri Reticulatae Pericarpium* sample.

### 2.4. UV Spectrophotometry

Ultraviolet-visible (UV-Vis) spectrophotometry is used to qualitatively and quantitatively analyze compounds by utilizing the absorption spectra of compounds that will absorb energy in the UV or visible region and undergo an electron energy leap. In addition to simple operation and fast analysis speed, UV-Vis has the advantage of greater sensitivity and selectivity [48].

In a study to determine whether *Pueraria lobata* could protect human umbilical vein endothelial cells (HUVECs) from apoptosis, Gao et al. used UV spectrophotometry and HPLC to determine the content of isoflavones in the ethanolic extracts of *Pueraria lobata*. Puerarin, daidzin and daidzein were found to be the major isoflavonoid components of *Pueraria lobata*, accounting for 84.94% of the extract [29]. Wang et al. isolated four known isoflavone analogs, a new isoflavone and a new flavone hydrate from *Pueraria lobata* (Willd.) ohwi, and their structures were characterized by IR, UV, HR-ESI-MS, 1D and 2D NMR spectroscopic methods [34]. Xie et al. measured the total flavonoids content of *Capsella bursa-pastoris* (L.) extract to 65.18±2.16% based on UV-VIS spectrophotometry [22]. Witkowska-Banaszczak et al. used NMR, UV spectroscopy and electrospray ionization tandem mass spectrometry (ESI-MS/MS) for the structural identification of 10 flavonoids in the extracts from the flowers of *Trollius europaeus* [31]. El Shoubaky et al. isolated a flavonoid from the acetone extract of marine red alga *Acanthophora spicifera* and identify the structure of the flavone compound by infrared, mass and UV spectroscopy. This flavonoid was confirmed to be apigenin and showed promising analgesic, anti-inflammatory and anti-proliferative activities [32]. Luteolin, a flavonoid widely occurring in natural plants, has a variety of activities including anti-inflammatory, cardioprotective and can interact with certain metals and biomolecules. Jomova et al. used UV-vis spectroscopy to characterize the interaction between Cu(II) and luteolin in their study of the effect of luteolin on DNA damage in the copper-catalyzed Fenton reaction [33].

### 2.5. Near Infrared Spectroscopy

Near-infrared (NIR) spectroscopy is a simple, fast, accurate and non-destructive technique that has been used in a variety of fields for process analysis and quality control in recent years [49,50,51]. Compared to the mid-infrared (MIR) range, the shorter NIR wavelengths increase the depth of penetration. NIR spectroscopy covers the wavelength range from 800 to 2500 nm and mainly records the spectral bands corresponding to the molecular vibrations of hydrogen bonds (e.g., C-H, N-H, O-H) to obtain the characteristic information of the hydrogen-containing groups in compounds [52]. Compared to IR spectroscopy, which requires the sample powder and KBr powder to be mixed and ground and pressed into tablets first, IR spectroscopy requires no sample preparation and will not produce any waste products [17]. However, NIR spectra usually rely on reference methods and need to be combined with chemometrics to build models [53].

Arslan et al. performed quantitative analyses of flavonoid components in black wolfberry by Fourier-transform near-infrared (NIR) spectroscopy combined with chemometric algorithms [34]. Wang et al. used near-infrared (NIR) spectroscopy to quantitatively monitor the content of flavonoid active ingredients in the water-ethanol extraction process of *Pueraria lobata* [35]. Betances-Salcedo et al. analysed the total contents of flavones and flavonols, flavanones and dihydroflavonols by using the methods of NIR methodology in 99 samples of propolis from Spain and Chile [36].

In general, herbal medicines contain a large number of active ingredients and other components that make spectroscopic measurements very complex, and it is difficult to achieve accurate measurements from NIR spectroscopy results alone. Therefore, multiple techniques are often used to obtain more complete and comprehensive information. UV-Vis detects only information about specific groups in the molecule, so UV-Vis cannot obtain information about all active compounds, but it is possible to get rid of the interference of solutions during spectroscopic analysis. In contrast, NIR is greatly affected by the solution, but more signals of the compound can be obtained. Combining NIR and UV-Vis, therefore, gives more comprehensive and effective information in a dual spectrum. Nie et al. developed a rapid method for the determination of flavonoid compounds (chrysin and galangin) in poplar propolis by means of visible and near-infrared spectroscopy (Vis-NIR) and a total of 114 propolis samples from China were analysed [37].

The effective extraction of Chinese medicinal ingredients is the primary premise behind identifying the structure and studying the biological activity, which directly determines the quality of the preparations in production and the actual clinical efficacy. Therefore, a reasonable extraction time is crucial, too long or too short an extraction time will affect the extraction yield. Therefore, a simple, rapid and efficient means of monitoring and analysis is required in the extraction process of herbal medicines.

In this context, Xu et al. developed a dual-spectrum portable spectrometer technology based on near-infrared (NIR) and ultraviolet-visible (UV-Vis) spectroscopy. With the spectral acquisition analysis, a quantitative analysis model of total flavonoids in *Pueraria lobata* was developed to enable online monitoring of the extraction process of flavonoid active compounds from *Pueraria lobata* [38]. In particular, they have chosen to use cylindrical cuvettes instead of traditional quartz cuvettes or fibre optic probes when measuring spectroscopy, making it easier to clean the solid residues produced during the extraction process, while reducing the cost of detection and facilitating its widespread application. The results showed that the dual-spectrum online monitoring system is simple to operate, has fast sampling speed, low cost and provides more comprehensive information than individual NIR or UV–Vis spectrum. It is a promising tool for the quantitative analysis of some traditional Chinese medicines with complex compositions. It should be noted, however, that if the compound component to be measured does not absorb in the UV–Vis region, then the dual-spectrum system will not be able to identify it.

### 2.6. Hyperspectral Imaging

Hyperspectral imaging (HSI) combines imaging and spectroscopic techniques so that spectral and spatial information about the sample can be obtained simultaneously. Compared to conventional analytical methods such as liquid chromatography, hyperspectral imaging is faster and more non-destructive and has been used to detect various compositions of samples, such as food [54], agriculture [55] and herbal medicine [56,57].

Near-infrared (NIR) spectroscopy generally collects spectral information from small sampling points and is unable to obtain the spectra of the entire sampled region; the final spectra of the sample are represented by the average spectra measured from multiple small sampling points. In hyperspectral imaging, a 3D data cube (x × y × λ) is created by combining two spatial (x; y) and one wavelength (λ) dimension, where images are collected as a function of wavelength [57,58,59]. Hyperspectral imaging has the advantage of acquiring spectral information from the entire sampling area within the hyperspectral images. As a result, hyperspectral imaging enables more representative spectral information to be obtained than near-infrared spectra acquired from a single point. In addition, based on the character that each pixel within the hyperspectral images has a spectrum, a distribution map can be formed to explore the composition distribution differences within and among samples [60,61,62].

Zhang et al. determined the total flavonoid content of dried black goji berries by near-infrared hyperspectral imaging (NIR-HSI) [39]. In the work of He et al. near-infrared hyperspectral imaging was used to determine the total polysaccharide and total flavonoid content of *Chrysanthemum morifolium* [40]. Compared to reference methods using UV–Vis spectroscopy for total polysaccharides and total flavonoids, hyperspectral imaging was more environmentally friendly, and more efficient in handling large numbers of samples and online predictions could be made for different *Chrysanthemum morifolium*.

## 3. Identification of Quality

Generally, the quality of herbs can be strictly influenced by the culture conditions, growth year, and geographic origin, thus resulting in significant differences in their medicinal quality and clinical efficacy [63]. Differences in culture conditions, such as light, temperature, and air humidity, can affect the number of secondary metabolites accumulated in herb plants, while the content of these active ingredients increases with the number of growth years. The composition and content of active compounds in the same herb may vary depending on the geographical origins [64]. Hence, the cultivation methods, growth years and geographical origin of herbs have attracted more and more attention from consumers. It is necessary to establish a reliable and effective analytical method for identification.

### 3.1. Cultivation Methods and Growth Years

Hai et al. collected a total of 320 samples of *Dendrobium huoshanense* (DHS), which were mainly divided into greenhouse cultivation and wild-like cultivation, each cultivation containing four growth years [65]. It is difficult to identify and distinguish between samples of *Dendrobium huoshanense* from different cultivation methods and growth years due to the high similarity of these samples and the limited information obtained from a single spectrum. Therefore, Hai et al. synthesized metallized tetraphenylporphyrin (ZnTPP) to obtain nano-effect near and mid-infrared spectroscopy by axial coordination, hydrogen bonds or electrostatic interactions with flavonoids, amplifying the differences in spectral signals of *Dendrobium huoshanense* samples. The nano-effect near and mid-infrared spectral data were then fused to obtain nano-effect feature fusion spectra, which successfully identified samples of *Dendrobium huoshanense* (DHS) with different growth years and cultivation methods, achieving 100% accuracy. Tetraphenyl zinc porphyrin (ZnTPP) is a metalloporphyrin formed by the introduction of Zn^2+^ into the cavity in the porphyrin ring. ZnTPP achieved complementary properties of both substances, with highly large π-conjugated properties and excellent optical properties [66,67]. The results showed that the addition of ZnTPP increased the peak difference, and the overlap of the nano-effect mid-infrared spectra was reduced compared to the original spectra. For the nano-effect NIR spectra, the peaks were more dispersed overall and the difference in spectral properties increased. It indicated that the nano-effect of ZnTPP amplifies the variability of the near and mid-infrared spectra.

### 3.2. Geographic Origin

NIR spectroscopy, with the advantages of simple, rapid, high efficiency and no sample preparation, has been a powerful analytical tool in use for identifying and analysing the geographical origin of food and herbal medicines [68]. Chen et al. collected a total of 250 raw material samples of notoginseng from four main geographical origins (Yunnan, Xizang, Guangxi and Guizhou provinces of China). The geographical origin of notoginseng was successfully identified by near-infrared spectroscopy [69].

However, for those herbs with complex and similar compositions, their NIR spectral peaks often overlap severely and some significant differences in the spectral peak cannot be obtained, and the classification accuracy is low. Therefore, the original NIR spectra are difficult to be used directly for the quality evaluation of herbal medicines.

At present, a large number of sensors based on nanomaterials are widely used to monitor food safety and authenticity due to their low cost, high sensitivity, and convenience [70,71,72]. As a very popular nanomaterial, carbon dots(CDs) have many merits, such as remarkable optical properties, excellent biocompatibility, good stability, and environmental friendliness, and they have been widely used in chemical sensors. Porphyrins possess a rigid macrocyclic structure, a large π-conjugation system, and various functional groups and substituents, enabling them to recognize multifarious molecules.

In order to solve this dilemma, Long et al. completed the identification of the geographical origin of lily using carbon dot-tetramethoxyporphyrin nanocomposite(CDs-TMPP)-based nano-effect near-infrared spectroscopy sensor [73]. Long et al. first collected a total of 120 lily samples from 12 different geographic origins in China and then collected nano-effect NIR spectra in the presence of CDs-TMPP. The results show that the nano-effect spectroscopy sensor method had better classification performance compared with the original NIR spectra, with 100% accuracy in identifying the geographical origin of the lily samples. The active components in the lily interact with CDs-TMPP through hydrogen bonding, electrostatic interaction, and steric hindrance, enhancing the differences in near-infrared spectroscopy peaks of lily samples from different geographical sources. In previous studies, Lv et al. developed a near-infrared spectroscopic sensor combined with porphyrin to realize the identification of dendrobes from 12 different geographical sources [74]. Compared with traditional NIR spectra, the NIR spectra with TMPP can obtain more characteristic information, which greatly improves the accuracy of model identification. After adding TMPP, the accuracy rate of NIR spectra reached 100%. The possible mechanism was that the π-π conjugated system and the methoxy groups of TMPP interact with the chemical components of dendrobes, which increase the specificity of NIR spectra.

## 4. Interaction Studies

Spectroscopy techniques can be used not only for quantitative and qualitative analysis, but also in combination with bioactivity analysis to study the interaction of flavonoids bound to biological macromolecules, including lipid membranes, human serum albumin, and hyaluronidase.

### 4.1. Interaction of Flavonoids with Membrane Lipids

The amphiphilic character of flavonoids allows them to intercalate into or bind with lipid bilayers. Many of the biological effects of flavonoids have been assumed to result from interactions with the membranes [75,76,77]. In previous studies, IR spectroscopy has been used extensively to elucidate the interactions between flavonoids and membrane lipids, but the conclusions drawn from IR spectroscopy about the way flavonoids are incorporated onto or into lipid bilayers are rather vague and are generally not far reaching [78,79]. The main reason lies in the fact that the actual state in which the flavonoid molecules are bound to lipids in a buffer solution is not known.

When there are interactions between flavonols and their surroundings, changes in molecular structure may occur, resulting in changes in the spectrum. Such structural changes such as structural rearrangements, conformational changes and deprotonation may give rise to hydroxyl and carbonyl vibrations. The vibrations of anyone hydroxyl group do not occur in isolation but are mixed with ring vibrations and other neighboring hydroxyl groups to form very complex vibrational patterns. The structural changes caused by the interactions do not remain in a narrow part of the spectrum, but actually affect the whole spectrum. This leads to very different spectra of structurally similar compound molecules. Therefore, it can hardly be satisfactorily to discuss the spectra of flavonoids in terms of characteristic vibrations of either the hydroxyl or carbonyl group. Baranović et al. performed the infrared spectroscopic analysis of seven flavones (flavone, 3-and 5-hydroxyflavone, chrysin, apigenin, fisetin and luteolin) and five flavonols (galangin, kaempferol, quercetin, morin and myricetin) and reported that hydroxyl and carbonyl vibrations in the interaction of these flavonoids with membrane lipids [80]. By monitoring spectral changes brought about by the interaction of flavonoids with membrane lipids it revealed structural details of how the flavonoids incorporate onto or into the lipid bilayer.

### 4.2. Interaction of Flavonoids with HSA

Human serum albumin (HSA), the most abundant protein in plasma, has the capability of binding reversibly to a large variety of drugs via its binding sites. After binding to human serum albumin, the drug compound is transported to various locations in the body for release. Therefore, the strength of the drug compound’s ability to bind with human serum albumin determines the therapeutic effect [81]. A large part of the general population is exposed to some flavonoids in their daily diet. Consequently, the risk of interaction between the binding levels of these flavonoids and human serum albumin is much higher. This interaction is likely to alter the binding of another drug to human serum albumin, thus affecting the pharmacological effect of another drug.

Quercetin (QUE) is one of the most abundant flavonoids in the human diet and is also the main active ingredient in many herbal medicines, with a variety of pharmacological effects on the human organism [82]. Diosmin (DIO), a flavonoid commonly found in citrus fruits, is the active component of many drugs, especially ones used in the treatment of various blood vessel disorders [83]. Catechins (CAT) are also common plant-derived flavonoids with anti-inflammatory and antioxidant potential and have a wide range of applications in the pharmaceutical and food industries. Tigecycline (TGC), is an antibiotic drug commonly used in clinical practice. Sovrlić et al. investigated the effect of flavonoids (catechins, quercetin and diosmin) on the binding of antibiotics (tigecycline) to HSA using multiple spectroscopic measurements, as well as their effects on the structure of the active site and the nature of interactions [84]. The formation of triple complexes of HSA–TGC–FLAVs with high binding affinity was demonstrated by UV–Vis absorption spectroscopy and fluorescence analysis. The conformational changes of HSA were analyzed by simultaneous fluorescence spectroscopy, Fourier transform infrared spectroscopy and circular dichroism, and it was found that the triple complex of HSA–TGC–FLAVs did not affect the microenvironment around the tryptophan (Trp) and tyrosine (Tyr) residues of HSA.

#### 4.2.1. UV–Vis Absorption Spectra

UV–Vis absorption spectroscopy, a convenient, rapid and effective technology, is commonly used to study protein-drug interactions and complex formation [85]. Red-shift and blue-shift changes observed in UV–Vis spectra can explain the interaction mode between drugs and proteins. UV–Vis spectral results showed that the absorption intensity of HSA- flavonoids (QUE, CAT and DIO) decreased as the concentration of TGC increased, demonstrating the formation of triple protein–drug–drug complexes between HSA, TGC and flavonoids (QUE, CAT and DIO) [84].

#### 4.2.2. Fluorescence Quenching Measurements

The presence of Trp and Tyr amino acids in the HSA structure provides HSA with fluorescent properties. This fluorescence property is sensitive to the microenvironment of the HSA molecule and when the local environment of the HSA molecule changes, such as protein denaturation or biomolecular binding, its fluorescence is quenched [86,87]. In previous studies, Matei et al. found that the fluorescence of HSA was quenched in a concentration-dependent manner upon interaction with kaempferol by fluorescence measurements [88]. Kaempferol, a natural flavonoid compound with a wide range of biological activities, is widely found in plants and foods. Therefore, the change in the fluorescence intensity of HSA can determine whether small molecules are bound to it, affecting the environment around the Trp and Tyr amino acid residues. The results showed the fluorescence quenching of HSA-flavonoids (QUE, CAT and DIO) in the presence of varying concentrations of TGC, indicating the formation of the HSA-TGC-flavonoids (QUE, CAT and DIO) complex [84].

#### 4.2.3. Synchronous Fluorescence Spectra

The synchronous fluorescence method allows information on the molecular environment in the vicinity of the Trp and Tyr fluorophores of HSA to be obtained and to determine whether polarity change around the microenvironment has occurred by measuring the position of its maximum emission wavelength. Synchronous fluorescence of HSA can provide characteristic information around tyrosine (Tyr) and tryptophan (Trp) residues when the scanning wavelength intervals (Δλ) are fixed at 15 nm and 60 nm, respectively [89]. The red or blue shift in the maximum fluorescence emission (wavelength) of HSA indicates enhanced hydrophilicity or hydrophobicity of the microenvironment around Tyr or Trp residues, respectively [84]. In the findings of Matei et al., no significant shifts in the position of the maximum emission wavelength were registered, but the fluorescence burst of Trp was stronger than that of Tyr, suggesting that the binding site of kaempferol to HSA is nearer to the Trp residue [88]. The results of the synchronous fluorescence spectra of the ternary HSA–TGC–flavonoids (QUE, CAT and DIO) systems showed no significant changes in the maximum emission wavelength of Tyr and Trp residues, indicating that the interaction of HSA-TGC with flavonoids does not affect the conformation of the micro-region of Tyr and Trp [84].

#### 4.2.4. Circular Dichroism Measurements

It is known that protein–ligand interactions can alter the secondary structure, resulting in changes in the protein conformation, which are reflected by the circular dichroism (CD) spectrum. The circular dichroism spectrum of HSA observed two negative minima at 208 and 222 nm, and represents α- helix structure transition of π → π^⁎^ and n → π^⁎^. In the findings of Matei et al., the percentage of α-helices decreased progressively upon binding of kaempferol to HSA, compared to free HSA molecules, indicating a degree of protein folding [88]. Sovrlić et al. found that the CD spectra for the ternary HSA-TGC-flavonoids (QUE, CAT and DIO) systems showed no significant change in signal and shape from the original spectra, indicating that the binding of HSA–TGC–flavonoids had a negligible effect on the secondary structure of the protein [84].

#### 4.2.5. Fourier Transform Infrared Spectroscopy (FT-IR)

The investigation of the secondary structure of HSA was performed using the FT-IR spectroscopic technique [88]. The protein amide I band at 1650–1654 cm^−1^ and amide II bands at 1548–1560 cm^−1^ are attributed to the secondary structure of all proteins. FT-IR spectra of HSA showed no significant difference between the ternary HSA-TGC-FLAV system and free HSA, indicating no conformational change in the HSA protein [84].

### 4.3. Interaction of Flavonoids with Hyaluronidase

It has been reported that there were several enzymes known to be involved in promoting inflammatory pathways, of which hyaluronidase (HAase) is one of the most important enzymes, cleaving hyaluronic acid in the extracellular matrix and improving the permeability of cell membranes and blood vessels [90]. During the development of inflammation, the level of HAase in the body increases dramatically. It has been found that some flavonoids exhibit strong anti-inflammatory effects while being able to inhibit the activation of HAase [91].

#### 4.3.1. Fluorescence Spectra

In the study by Zeng et al., the interaction between eight flavonoids(apigenin, luteolin, keampferol, quercetin, morin, naringenin, daidzein, genistein) and HAase was investigated by fluorescence spectroscopic and molecular modeling methods [90]. The results showed that the eight flavonoids formed flavonoid–HAase complexes mainly by binding to HAase interactions through electrostatic forces, hydrophobic interactions and hydrogen bonding. According to synchronous and three-dimensional fluorescence spectra, the presence of flavonoids significantly altered the microenvironment and conformation of HAase, leading to reduced enzyme activity.

Li et al. studied the binding of three flavonoids(baicalin, liquiritin and isoliquiritigenin), extracted from *Scutellaria baicalensis Georgi* and *Glycyrrhiza uralensis* to hyaluronidase by steady state fluorescence, time-resolved fluorescence and circular dichroism (CD) spectroscopy [92]. The results of the fluorescence spectra showed that when baicalin and liquiritin were bound to HAase, the strongest fluorescence emission peak of HAase was red-shifted and fluorescence quenching was stronger. When isoliquiritigenin interacted with HAase, it acted as quencher to decrease the fluorescence intensity of HAase with no significant change in the position of the emission peak. The fluorescence quenching mechanism of HAase by the three flavonoids is a static quenching procedure.

#### 4.3.2. Synchronous Fluorescence Spectra

Synchronous fluorescence spectra present information about the molecular microenvironment in the vicinity of the fluorophore by measuring the emission wavelength shift. The wavelength interval (Δλ) between the excitation wavelength and the emission wavelength is fixed individually at 15 and 60 nm, which gives the characteristic information of tyrosine (Tyr) or tryptophan (Trp), respectively.

Synchronous fluorescence spectra showed that with the addition of baicalin, the maximum emission wavelengths of Trp- and Tyr- are both observed to have a redshift, indicating that the polarity around Trp-and Tyr-residues increases and the hydrophobicity decreases. For the liquiritin–HAase system, the maximum emission wavelength of Trp-residues has an obvious red shift, but that of Tyr- has no obvious change. The results suggested that the interaction of liquiritin with HAase increases the polarity and decreases the hydrophobicity around Trp-residue, but has no effect on the microenvironment around Tyr-residue. For the isoliquiritigenin–HAase system, the synchronous fluorescence peaks of Trp- and Tyr- do not change significantly, indicating that the microenvironment of Trp-and Tyr-is not disturbed by isoliquiritigenin [92].

#### 4.3.3. Circular Dichroism

Circular dichroism (CD) is usually executed to investigate the secondary structural changes of protein because of its accuracy and sensitivity [93]. The results of CD spectroscopy showed that the binding of flavonoids(baicalin, liquiritin and isoliquiritigenin) with HAase leaded to changes in the secondary structure of HAase with an increase in the α-helix content in HAase.

## 5. Chemometrics

Due to the complex composition of herbal raw materials, the obtained spectra are difficult to identify, have severe overlaps of spectrum bands and contain a lot of useless information, which brings a great challenge for the identification of spectral information. Therefore, it is necessary to analyse the spectral information of the samples by chemometric methods in order to obtain valid spectral information, improve the selectivity of fluorescence spectroscopy and achieve rapid multi-component analysis. Table 1 has summarised the chemometric methods used for the spectroscopic analysis of medicinal and food homology materials. 

### 5.1. PLS-DA Analysis

Partial least squares discriminant analysis (PLS-DA) is a very common classification method that is used in various fields of analysis [94,95,96]. PLS-DA is based on the classical PLS regression algorithm, which combines PLS regression with discriminant analysis to look for latent variables (LV) with a maximum covariance with the dependent Y variable. The number of LVs is usually determined based on the optimal correct classification rate of the cross-validation procedure [97]. The dependent variable Y is a dummy matrix composed of binary values, with the one-hot encoding used to represent the class belonging of the samples. In dummy matrix Y, a value of 1 means that the sample belongs to a specific class and 0 means the opposite. However, the estimated response values of PLS regression models are usually not exactly equal to 0 or 1. When the estimated response value for a sample is closer to 1, the sample is considered to belong to the corresponding class, and the opposite is true when it is closer to 0.

The model based on the traditional raw fusion spectrum is poor, resulting in low accuracy in the training and prediction sets. It means that traditional spectra struggle to identify DHS from different cultivation years. To enable feature fusion spectra of DHS samples of different growth years to be identified by classification, PLS-DA was used to build a PLS-DA model based on nano-effects feature fusion spectra. Of these, 70% of the DHS samples were classified as the training set and 30% were classified as the prediction set, with the training and prediction sets proceeding by random classification. The results showed that the PLS-DA model based on nano-effects feature fusion spectroscopy can achieve accurate discrimination of DHS samples of different growth years with 100% accuracy [65].

Long et al. used partial least squares discriminant analysis (PLS-DA) to identify the geographical origin of the lily based on the collected nano-effect NIR spectroscopy, achieving 100% classification accuracy [73]. Lan et al. collected nanomaterial-based fluorescence splicing spectra of *Citri Reticulatae Pericarpium* samples and achieved 100% sample species identification and 98.04% storage year identification by partial least squares discriminant analysis [28]. In order to classify dendrobe of different geographical origins, Lv et al. used partial least squares to process the NIR spectral data of dendrobe samples and achieved 100% accuracy in identifying the origin of the samples [74]. Arslan et al. used partial least squares(PLS) to establish the quantitative analysis models of the total flavonoid content and other components in black goji berries based on near-infrared fourier transform spectroscopy [34]. Yin et al. developed a quantitative prediction terahertz time-domain spectroscopy model of the ternary flavonoid mixtures (genistein, naringenin, daidzein) by means of partial least squares regression, achieving simultaneous prediction of the concentrations of these three analytes [26]. Wang et al. used partial least squares regression (PLSR) to develop a calibration model for near-infrared spectroscopy that could be used to rapidly monitor the concentration of isoflavone compounds during *Pueraria lobata* extraction [35]. Betances-Salcedo et al. used a modified partial least squares (MPLS) regression method to develop a data calibration model to evaluate the NIR spectral data of 99 propolis samples, which well quantified the composition of flavonoids and flavonols, flavanones and dihydroflavonols in propolis [36]. The MPLS model calculates and standardized the NIR residuals for each factor and wavelength and is typically more stable and accurate than the standard PLS algorithm.

### 5.2. OPLS-DA Analysis

Orthogonal partial least squares discriminant analysis (OPLS-DA) is a very effective supervised analysis method that is commonly used to deal with classification and discrimination problems [98]. The orthogonal partial least squares discriminant analysis (OPLS-DA) adds a positive exchange algorithm to the partial least squares discriminant analysis (PLS-DA), which can filter out signals that are not relevant in the model’s classification matrix [99]. Therefore, the OPLS-DA model is able to maximize the classification differences between groups and can better identify differences in chemical composition between groups compared to the PLS-DA model. The classification indicators of the OPLS-DA model include accumulated explanatory power parameters (R2X, R2Y) and predictive ability parameters (Q2) [100]. Among them, R2X and R2Y respectively represent the percentage of X and Y matrix information that the OPLS-DA classification model can explain, and Q2 is calculated through cross-validation to evaluate the predictive ability of the OPLS-DA model.

The closer these indicators are to 1, the better the OPLS-DA model fits the data and predictive power. When these indicators are greater than 0.5 the model is considered to have good results. In the results of Hai et al., an OPLS-DA model for nano-effects feature fusion spectroscopy was developed with model parameters including R2X, R2Y and Q2 are both greater than 0.9, indicating that the model has good goodness-of-fit and prediction ability to successfully distinguish between different cultivation mode of DHS samples [65].

### 5.3. VIP Value Analysis

The variable importance for the projection (VIP) value describes the importance of each variable in the models, and variables with a VIP score above 1 will be considered to contribute significantly to the PLS-DA model, thereby classifying and screening important spectral information [101]. Screening valid information through VIP can improve the model classification accuracy for subsequent chemometric analysis.

In order to improve the accuracy of identifying the cultivation methods and growth years of *Dendrobium huoshanense* (DHS), Hai et al. processed the spectral data by chemometrics after obtaining nano-effect near-infrared spectroscopy and nano-effect mid-infrared spectroscopy of 320 DHS samples [65]. After screening by variable importance for the projection (VIP greater than 1), multivariate data extraction and integration are performed to obtain the feature vectors of the fusion spectra. The feature vectors were then combined with partial least squares discriminant analysis (PLS-DA) and orthogonal partial least squares discriminant analysis (OPLS-DA) to identify DHS in different growth years and cultivation methods.

### 5.4. Data Fusion Strategy

A data fusion strategy is the integration of data from all sources to provide complementary data on the overall chemical signature [102]. It can be divided into three levels: low, medium and high. Compared with single data analysis, the data fusion strategy provides more efficient and accurate chemometric characterization, which is advantageous for tracing geographical origin [103] and quality identification [104]. Low-level data fusion is the concatenation of signals from different analysis instruments to form a new matrix where the rows represent the number of samples to be analysed and the columns represent the signal variables. Medium-level data fusion extracts the desired features from the signals of different analysis instruments and then concatenated these features into a new matrix for multivariate classification analysis. High-level data fusion calculates the classification results for each dataset separately and then finishes the combined analysis by assigning ratios based on discriminant accuracy.

The original data of the DHS samples were screened with VIP to retain nearly half of the important variables [65]. The extracted feature vectors were then subjected to data fusion to obtain the NIR-MIR fusion spectra and nano-effect NIR-MIR fusion spectra, which contain more comprehensive and valid information compared to the original spectra.

### 5.5. Sampling Error Profile Analysis (SEPA) Method

The sampling error profile analysis (SEPA) method is based on the Monte Carlo sampling(MCS) strategy and error profile analysis and can be used in outlier detection, CV, pretreatment method and wavelength selection, and model evaluation [105]. Multiple sub-models and their resulting sub-errors were obtained from the MCS. Error distribution analysis was performed on these sub-errors, from which the median, variance and bias of the errors were estimated. In spectral analysis, the choice of the number of latent variables (LV) and the evaluation of the model are essential for the optimization of the spectral analysis model. Cross validation (CV) is a commonly used method for selecting the number of LVs and can be used for model optimization. In both model optimization and evaluation, error analysis of statistical models is required. The median is more robust and can evaluate the model more accurately instead of the mean. The variance and bias of the model determine the predictive power of the model. As the complexity of the model increases, the bias of the model becomes smaller and the variance higher, but the generalization ability decreases and the prediction error becomes more significant, resulting in over-fitting of the data. Therefore, an optimal model should have a good complexity and a small prediction error, which means that the model is accurate and robust. Meanwhile, after the data have been fitted, the parameters selected in the model need to be tested for adequacy. The SEPA method can make the model more predictive and stable.

Xu et al. established a SEPA-PLS model based on the near-infrared (NIR) and ultraviolet–visible (UV–Vis) dual spectra of *Pueraria lobata* and achieved online quantitative monitoring of total flavonoids during the extraction of *Pueraria lobata* with good accuracy and precision [38]. SEPA-PLS models were developed using individual near-infrared (NIR) and ultraviolet-visible (UV–Vis) spectra from the extraction process of *Pueraria lobata*. Possible outliers were screened by error analysis. Finally, NIR and UV–Vis spectral data were fused to construct the dual-spectrum model.

### 5.6. Principal Component Analysis

Principal Component Analysis (PCA) is a classical multivariate statistical and data processing method that is widely used in a variety of analytical fields [106,107]. PCA can be used to extract feature variables, reduce the dimensionality and removes overlapping information from the data set without reducing the variance, and highlights the characteristics of the data through principal components. PCA is an orthogonal transformation method that changes the original correlated variables to uncorrelated components, which are named principal components (PCs), namely, a linear combination of the original variables. PCs are arranged in descending order of variance. When the cumulative variance contribution rate is more than 85%, these PCs can be considered to be able to replace the original data set [108].

Yin et al. collected terahertz time-domain spectroscopy (THz-TDS) of 10 common flavonoids, including baicalein, baicalin, apigenin, quercetin, naringenin, hesperetin, daidzein, genistein, puerarin, and gastrodin. Then the THz-TDS of these flavonoids were qualitatively identified and quantitatively analysed by chemometric methods, including principal component analysis (PCA), support vector machine (SVM), partial least squares regression (PLSR) and artificial neural networks (ANN) [26]. They performed a PCA method of the THz-TDS data of all samples and extracted the top five principal components representing the important information of the original data based on the three-dimensional score graph.

### 5.7. Support Vector Machine

Support vector machine (SVM) is a promising classification and regression technique for solving linear and non-linear multivariate calibration problems with excellent generalization capabilities [109]. Compared with other statistical methods, SVM does not require a large number of training samples for modeling [108]. Generally, PCA is combined with SVM techniques in order to improve the prediction accuracy of classification models.

Yin et al. used the THz-TDS spectral feature variables extracted by principal component analysis (PCA) as input variables of SVM to classify and identify 10 flavonoids. The model had 100% classification accuracy compared to the original spectral results [26]. Yan et al. performed quantitative analyses of three structurally similar flavonols by terahertz spectroscopy combined with partial least squares regression (PLSR) and least squares support vector machine (LS-SVM) [31]. The LS-SVM model demonstrated better results compared to the PLSR model for myricetin, quercetin, and kaempferol, respectively. He et al. used partial least squares (PLS) and least squares support vector machine (LS-SVM) to build prediction models and combined with near-infrared hyperspectral imaging to determine the total polysaccharides and total flavonoids content in *Chrysanthemum morifolium*, and obtained good prediction results [40]. Zhang et al. performed feature extraction by principal component analysis (PCA), developed partial least squares (PLS) and least squares support vector machine (LS-SVM) models, and used near-infrared hyperspectral imaging (NIR-HSI) techniques to determine the total flavonoid content in black goji berries [39].

### 5.8. Artificial Neural Network

Artificial neural network (ANN) is a commonly used non-linear econometric method, often used to solve machine learning problems such as regression and classification [110]. ANN is based on the operating principles of biological nerve cells and uses mathematical expressions to simulate the signal transmission between neurons, thus constructing interconnected hierarchical artificial neural networks. In spectral analysis, spectral data are introduced into the ANN model as an input layer and the output layer is the predicted result. The performance of the model is evaluated by the coefficient of determination and root mean square in the calibration (RMSEC) and the prediction set (RMSEP) [111]. Yin et al. used ANN regression models combined with THz-TDS for the quantitative detection of ternary mixtures of flavonoids (genistein, naringenin, daidzein) and showed good predictions [26]. Nie et al. used visible and near-infrared spectroscopy (Vis–NIR) combined with several chemometric models, including partial least squares (PLS), artificial neural networks (ANN), multiple linear regression(MLR) and least square-support vector machine (LS-SVM), to perform a rapid determination of the content of chrysin and galangin in poplar propolis. Among them, the ANN model achieved the best results [37].

### 5.9. Soft Independent Method of Class Analogy

The soft independent method of class analogy (SIMCA) is a classical class modeling technique that incorporates principal component analysis (PCA) to reduce the dimensions of the spectral data and provides a high dimensional variations classification [112]. The SIMCA model contains a collection of mutually independent PCA datasets. The training set is modeled by PCA and new samples can be fitted to the model and classified according to their similarity or dissimilarity to the training set. Chen et al. used partial least squares discriminant analysis (PLSDA) and soft independent modeling of class analogy (SIMCA) to construct the discriminant models that combined NIR spectral data to classify 250 notoginseng samples from different geographical origins, and the models achieved 100% sensitivity and 100% specificity on both the training and test sets [69].

## 6. Conclusions

The development and application of natural products have always been an area of significant interest to researchers. The abundant pharmacological activities of natural flavonoids demonstrate their adequate potential for future development and application in therapeutic drugs, functional foods and cosmetic additives. Phytochemical and pharmacological studies of medicinal and food homology materials are becoming increasingly attractive because of their combination of medicinal and food health effects. Nevertheless, to date, there are few reviews on the application of spectroscopic analysis of medicine and food homology flavonoids. This paper reviewed common spectroscopic methods applied to the analytical study of flavonoid components in medicinal and food homology materials, including qualitative and quantitative analysis of the compound structure and content, identification of herbal cultivation methods, growth years and geographical origin, interaction studies when combined with biomolecules, and chemometric methods used in combination with spectroscopic techniques.

Nuclear magnetic resonance (NMR) spectroscopy is a common and effective method for accurately identifying the chemical structure of multi-component complex samples. However, NMR has limited applicability in other analytical applications and is expensive. Terahertz spectroscopy is a very powerful technique that has emerged in recent years and has the advantages of being fast, safe and non-destructive, enabling the effective identification of structurally similar biomolecules. However, terahertz spectroscopy also has limitations in terms of limited penetration, scattering effects and limited sensitivity, and is very costly. Fluorescence spectroscopy is a sensitive, simple and rapid detection technique that is commonly used for quality control of food products and monitoring and analysis of the environment. In recent years, as nanotechnology becomes more sophisticated, various new nano-fluorescent sensor-based fluorescence spectroscopy has found more applications in quality assessment. Near-infrared (NIR) spectroscopy is a simple, fast, accurate, non-destructive and will not produce waste products technique that has been used in a variety of fields for process analysis and quality control in recent years. However, NIR spectra usually rely on reference methods and need to be combined with chemometrics to build models. In addition to simple operation and fast analysis speed, UV–Vis has the advantage of greater sensitivity and selectivity. UV–Vis detects only information about specific groups in the molecule, so UV–Vis cannot obtain information about all active compounds, but it is possible to get rid of the interference of solutions during spectroscopic analysis. In contrast, NIR is greatly affected by the solution, but more signals of the compound can be obtained. Combining NIR and UV–Vis, therefore, gives more comprehensive and effective information in a dual spectrum. Hyperspectral imaging (HSI) combines imaging and spectroscopic techniques so that spectral and spatial information about the sample can be obtained simultaneously. In addition, hyperspectral imaging enables more representative spectral information to be obtained than near-infrared spectra acquired from a single point. Compared to conventional analytical methods such as liquid chromatography, hyperspectral imaging is faster and more non-destructive.

A growing number of flavonoid components of medicinal and food homology materials have been isolated, identified and studied, based on the development of various techniques of spectroscopic analysis and other analytical methods. Nevertheless, continuous efforts are still needed to develop more analytical techniques with development potential to ensure quality control of medicinal and food homology materials and further clarify the potential molecular mechanisms. This will provide favorable conditions for better product development and market application of medicinal and food homology materials. This review may contribute to a rapid understanding of the application of spectroscopic techniques to flavonoid components in medicinal and food homology materials and provides an important reference for the research and development of flavonoids in medicinal and food homology materials.

## Figures and Tables

**Table 1:** Sources, flavonoid composition, spectroscopy techniques and chemometrics of medicinal and food homology materials.

Sources	Flavonoid Types	Compound Name	Analysis Technique	Purpose	Chemometrics	Year	References
Platycladi Cacumen	21	Myricitrin; quercitrin; afzelin; amentoflavone	NMR	Chemical structures	OPLSR	2022	[19]
Glycyrrhiza uralensis	22	Apigenin 6-C-α-L-rhamnoside-8-C-(6′-(3-hydroxy-3-methylglutaroyl)-β-D-glucoside); apigenin 6-C-α-L-glucoside-8-C-(6′-O-(3-hydroxyl-3-methylglurtaroyl)-β-D-glucoside); apigenin 6-C-α-L-arabinoside-8-C-(6′-O-(3-hydroxyl-3-methylglurtaroyl)-β-D-glucoside)	NMR	Chemical structures	OPLS-DA	2022	[20]
Astragalus membranaceus	14	7-β-D-glucopyranosyloxy-astrapterocarpan; (3R,4R)-7-(2-O-β-erythro-D-apiofuranosyl-β-D-glucopyranosyloxy)-3′,4′-dimethoxyl-pterocarpan	NMR	Chemical structures	\	2022	[21]
Capsella bursa-pastoris(L.)	4	Isoorientin; isoorientin-2″-O-α-L-arabinopyranosyl; isoorientin-2″-O-α-L-xylose; kaempferol-3-O-β-D-glucoside,	NMR; UV-Vis	Chemical structures; Quantitatively analyse	\	2021	[22]
Glycyrrhiza uralansis	10	Diosmetin; echinatin; Licofuranol A; licofuranol B; calycosin; luteolin; scopoletin; glycypytilbene B,	NMR; UV-Vis	Chemical structures	\	2019	[23]
Radix puerariae	2	Daidzein; puerarin	NMR	NMR chemical shifts; NMR shielding parameters	\	2019	[24]
Psoralea corylifolia L.	62	Bavaisoflavone; bavachinone; bavaflavone	NMR; Circular dichroism spectra	Chemical structures	\	2022	[25]
Flavonoids	10	Baicalein; baicalin; apigenin; quercetin; naringenin; hesperetin; daidzein; genistein; puerarin; gastrodin	Terahertz time-domain spectroscopy(THz-TDS)	Qualitative identification; quantitative analysis	PCA; SVM; PLSR; ANN	2020	[26]
Flavonols	3	Myricetin; quercetin; kaempferol	Terahertz spectroscopy	Qualitative identification; quantitative analysis	PLSR; least squares SVM	2018	[27]
Citri reticulatae pericarpium	6	Nobiletin; sinensetin; 3,5,6,7,8,3′,4′-heptamethoxyflavone; tangeretin; hesperidin	Fluorescence spectroscopy	Quality evaluation; identify storage year	PLS-DA	2020	[28]
Pueraria lobata	3	Puerarin, daidzin, daidzein	UV	Quantitative analysis	\	2016	[29]
Pueraria lobata	6	Lobatflavate, 3S,4R-tuberosin, daidzein, puerarin, daidzin, ononin	IR; UV; NMR	Chemical structures	\	2017	[30]
Trollius europaeus	10	Orientin; isoorientin; vitexin; isovitexin; orientin 2′-O-β-arabinopyranoside; orientin 2′-O-β-glucopyranoside; vitexin 2′-O-β-arabinopyranoside; vitexin 2′-O-β-galactopyranoside	NMR; UV; MS	Chemical structures	\	2018	[31]
Marine red alga acanthophora spicifera	1	Apigenin	IR; UV; MS	Chemical structures	\	2016	[32]
Flavonoid	1	Luteolin	UV-vis	Chemical structures	\	2021	[33]
Black wolfberry	Total flavonoids	Rutin	NIR	Quantitative analysis	PLS	2018	[34]
Pueraria lobata	Total isoflavonoid	Puerarin; daidzin	NIR	Quantitative analysis	PLS	2014	[35]
Propolis	Total flavones; flavonols; flavanones	Quercetin; rutin; pinocembrin	NIR	Quantitative analysis	PLS	2017	[36]
Poplar propolis	2	Chrysin; galangin	Vis-NIR	Quantitative analysis	PLS; ANN; MLR; LS-SVM	2013	[37]
Pueraria lobat	Total flavonoids	Puerarin	NIR; UV-Vis	Quantitative analysis	SEPA-PLS	2022	[38]
Black goji berries	Total flavonoids	Quercetin	Near-infrared hyperspectral imaging (NIR-HSI)	Quantitative analysis	PCA; PLS; LS-SVM	2020	[39]
Chrysanthemum morifolium	Total flavonoids	Rutin	NIR-HSI	Quantitative analysis	PLS; LS-SVM	2018	[40]

Notes: Orthogonal partial least squares regression (OPLSR); Partial least squares (PLS); Orthogonal partial least squares discriminate analysis (OPLS-DA); Principal component analysis (PCA); Support vector machine (SVM), Partial least-squares regression (PLSR); Artificial neural network (ANN); Multiple linear regression (MLR); Sampling error profile analysis (SEPA).

## Data Availability

Not applicable.
